# Efficacy of three-dimensionally printed polycaprolactone/beta tricalcium phosphate scaffold on mandibular reconstruction

**DOI:** 10.1038/s41598-020-61944-w

**Published:** 2020-03-18

**Authors:** Sanghoon Lee, Dami Choi, Jin-Hyung Shim, Woong Nam

**Affiliations:** 1 Department of Oral and Maxillofacial Surgery, Yonsei University College of Dentistry, 50-1, Yonsei-ro, Seodaemoon-gu Seoul, 03722 Republic of Korea; 2 Department of Oral and Maxillofacial Surgery, Jukjeon Dental Hospital, College of Dentistry, Dankook University, 152, Jukjeon-ro, Suji-gu, Yongin-si, Gyeonggi-do 16890 Republic of Korea; 3 Research Institute, T&R Biofab Co. Ltd.,237 Sangidaehak-Ro, Siheung-si, Gyeonggi-do 15073 Republic of Korea; 4 Department of Mechanical Engineering, Korea Polytechnic University, 237 Sangidaehak-Ro, Siheung-si, Gyeonggi-do 15073 Republic of Korea

**Keywords:** Dentistry, Dentistry, Preclinical research, Preclinical research

## Abstract

It has been demonstrated that development of three-dimensional printing technology has supported the researchers and surgeons to apply the bone tissue engineering to the oromandibular reconstruction. In this study, poly caprolactone/beta tricalcium phosphate (PCL/β-TCP) scaffolds were fabricated by multi-head deposition system. The feasibility of the three-dimensionally (3D) -printed PCL/β-TCP scaffolds for mandibular reconstruction was examined on critical-sized defect of canine mandible. The scaffold contained the heterogeneous pore sizes for more effective bone ingrowth and additional wing structures for more stable fixation. They were implanted into the mandibular critical-sized defect of which periosteum was bicortically resected. With eight 1-year-old male beagle dogs, experimental groups were divided into 4 groups (n = 4 defects per group, respectively). (a) no further treatment (control), (b) PCL/β-TCP scaffold alone (PCL/TCP), (c) PCL/β-TCP scaffold with recombinant human bone morphogenetic protein-2 (rhBMP-2) (PCL/TCP/BMP2) and (d) PCL/β-TCP scaffold with autogenous bone particles (PCL/TCP/ABP). In micro-computed tomography, PCL/TCP/BMP2 and PCL/TCP/ ABP groups showed significant higher bone volume in comparison to Control and PCL/TCP groups (P < 0.05). In histomorphometric analysis, a trend towards more bone formation was observed in PCL/TCP/BMP2 and PCL/TCP/ABP groups, but the results lacked statistical significance (P = 0.052). Within the limitations of the present study, 3D-printed PCL/β-TCP scaffolds showed acceptable potential for oromandibular reconstruction.

## Introduction

Since the introduction of microvascular free tissue transfer, vascularized bone graft (VBG) has become the treatment of choice for oromandibular reconstruction^1,2^. However, microvascular free tissue transfer might present surgical results with large variation depending on the surgeon’s skill and experience. It also requires considerable hospital resources including two surgical teams, an intensive care unit and prolonged hospital stay. Furthermore, donor-site morbidity may lead to important aesthetic, functional, and psychosocial issues for patients. To overcome the heavy burden of free tissue transfer, maxillofacial surgeons have turned to the field of tissue engineering for reconstruction^3–5^, of which potential advantages may include more customized reconstruction, shortened operation time, rapid recovery and minimization of donor-site morbidity^2^.

In recent years, the development of three-dimensional printing (3DP) technology for tissue engineering has contributed to increased speed and precision from planning to operation in maxillofacial reconstruction^6^. 3DP technology has been researched in manufacturing scaffolds with growth factor in conjunction with a surgical guide and template. It is capable of not only reproducing the individual anatomical complexity of a maxillofacial defect but also optimizing functions and mechanical properties through the combination of various materials, stem cells and growth factors^4,7,8^. Several *in vivo* and *in vitro* studies involving 3DP, particularly using a polycaprolactone/β-tricalcium phosphate (PCL/β-TCP) combination, have shown the feasibility of scaffolds^9,10^. Finally, although clinical application of 3DP in maxillary reconstruction has been reported in the Republic of Korea^11,12^, to the best of our knowledge, there are few studies using 3D-printed PCL/β-TCP scaffolds in mandibular reconstruction with a large defect model.

In this study, we suggest a novel design of PCL/β-TCP scaffolds for mandibular reconstruction. The 3D-printed PCL/β-TCP scaffolds were successfully fabricated by 3DP technology using a multi-head deposition system (MHDS). We assess the feasibility of 3D-printed PCL/β-TCP scaffolds for a critical-sized defect (CSD) of canine mandible.

## Material and Methods

### Experimental animals and materials

This study was designed as a controlled preclinical study involving eight male, adult beagle dogs. The dogs were aged 12–15 months and with a mean body weight of 12.5 kg. They had no systemic disease and showed intact dentition and healthy maxillofacial appearance. Each animal was maintained in an individual cage under standard laboratory environment and were provided with a prescription diet (a/d Urgent Care, Hill’s, Topeka, KS, USA) by a veterinarian and a standard laboratory pellet diet (Purina Canine LabDiet, Cargill, Dangjin, Republic of Korea). The animal selection, acclimatization, surgical protocols and management were approved by the Institutional Animal Care and Use Committee of Yonsei University Health System, Seoul, Korea (IACUC No. 2017-0326) and performed in accordance with the relevant guidelines and regulations.

### Design of PCL/β-TCP scaffold

**Figure 1:**
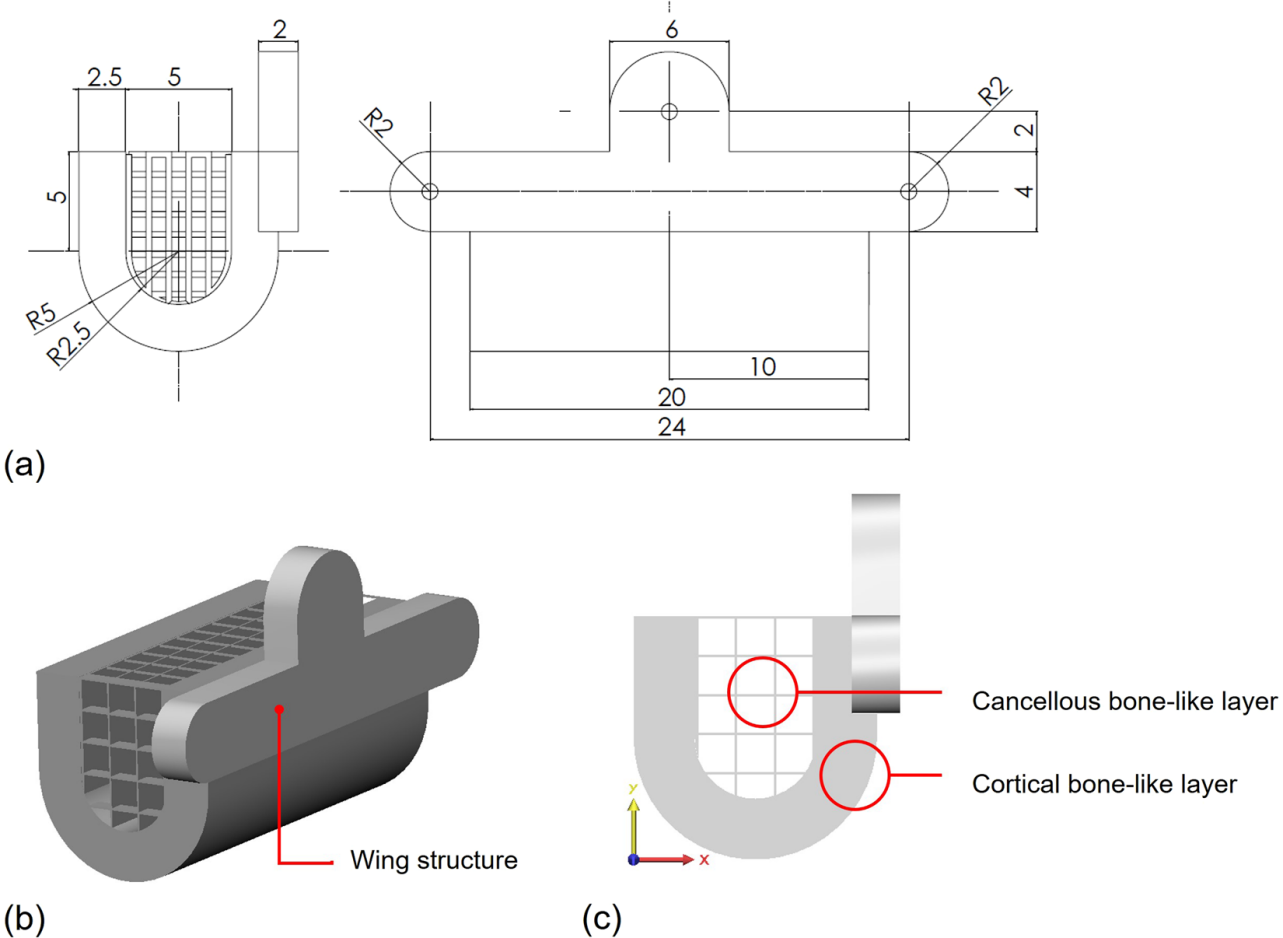
Design of 3D-printed PCL/ β-TCP scaffolds. (**a**) dimension, (**b**) wing structure for screw fixation, (**c**) different porosities.

**Figure 2:**
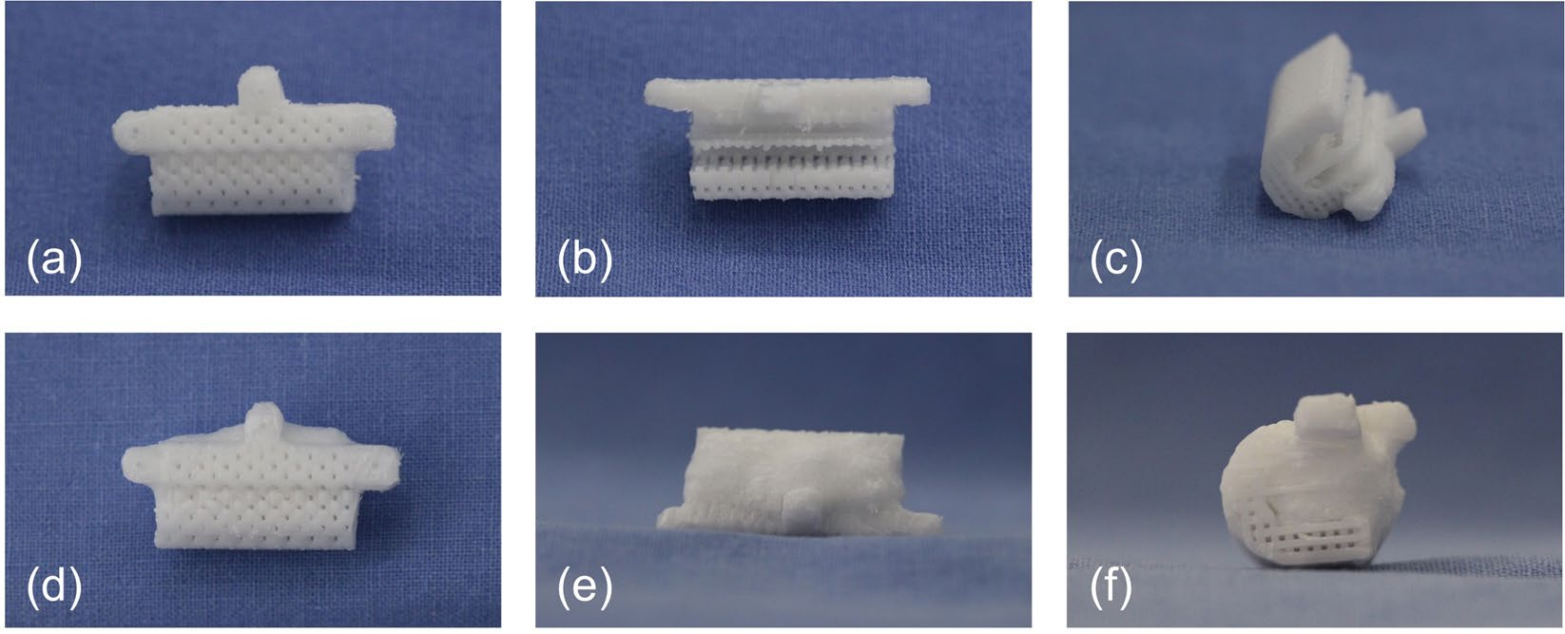
Morphology of 3D-printed PCL/ β-TCP scaffolds. PCL/β-TCP scaffold (**a**), sagittal view; (**b**), axial view; (**c**) coronal view) and PCL/β-TCP scaffold loaded with rhBMP-2 (**d**), sagittal view; (**e**), axial view; (**f**) coronal view).

For immobilization of scaffolds in mandibular defect, annexed wing structures containing three screw holes were designed for fixation. Wings are built up on three interfaces with residual bone (mesial, distal and alveolus) (Fig. 1a,b). The scaffold structure consists of two layers of different density (Fig. 1c). The external layer, comparable to the cortical bone, is composed of a dense layer with high porosity (70–75%) and pore size greater than 300 μm, appropriate for maintaining the strength of scaffold and *in vivo* vascularization. The internal layer, comparable to the cancellous bone, is composed of a loose grid of which diameter is greater than 600 μm for rapid bone regeneration from residual bone (Fig. 2a,b,c).

### Fabrication PCL/β-TCP scaffold using 3DP technology

**Figure 3:**
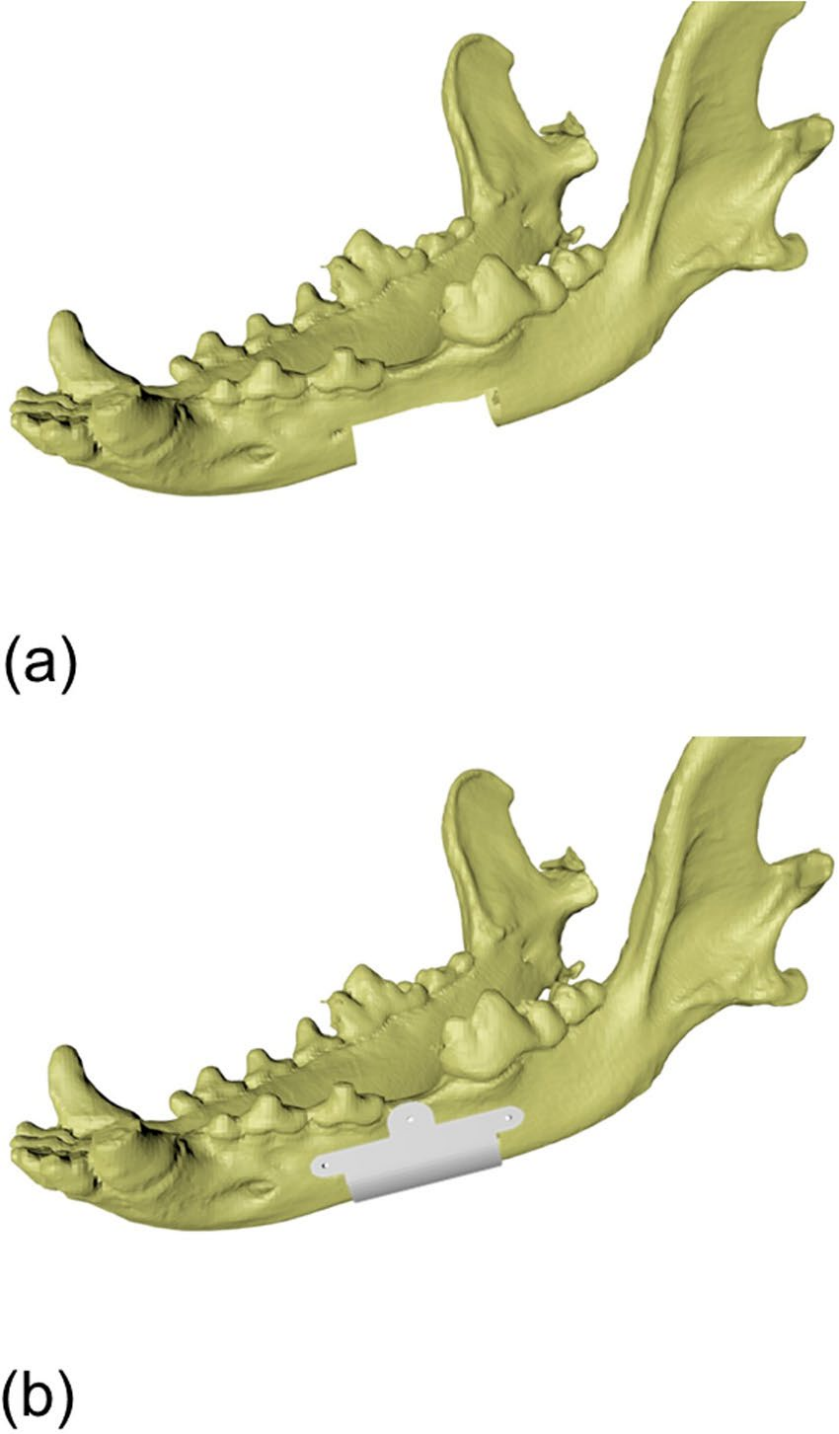
Modeling process of 3D-printed PCL/ β-TCP scaffolds. (**a**) 20 × 10 × 10 mm critical-sized defect was prepared, (**b**) 3D-printed scaffold was designed.

PCL (PC 12, Corbion Purac, Gorinchem, Netherlands) and β-TCP (average diameter, 100 nm; 7758-874, Premier Biomaterials, Tipperary, Ireland) were blended with a melting process. PCL was placed in a glass container and melted by heating for 15 min at 110 °C. After powdered β-TCP was added, PCL and the powdered β-TCP mixture were blended by hand for 10 min. The PCL/β-TCP mixture was transferred to a 10 ml steel syringe in the MHDS and maintained at 120 °C. MHDS was operated using computer-aided manufacturing software. The PCL/β-TCP material was extruded and stacked 4 times. The line width, pore size, and line height within the scaffolds were 300, 400, and 100 μm, respectively. The scaffolds had a rectangular pore architecture and a porosity of 57% as determined by 3D modeling software (3-Matic Research 9.0, Materialise, Leuven, Belgium) (Fig. 3). Pores were completely interconnected. The scaffolds were freeze-dried at −85 °C for 24 hours, then sterilized under a 450 W UV lamp for 4 hours.

The recombinant human bone morphogenetic protein-2 (rhBMP-2) loaded scaffolds were prepared for the PCL/TCP/BMP2 group as follows: 50 μg/ml rhBMP-2 solution (Cowellmedi, Busan, Republic of Korea) was mixed in 3% collagen solution (MS Biotech, Republic of Korea). 3D-printed PCL/β-TCP scaffold was immersed in the mixed solution. For penetration of mixed solution to the internal pores of scaffold, the mixed solution containing scaffold was centrifuged at 1000 rpm. The rhBMP-2/collagen-infiltrated scaffold was then freeze-dried at −85 °C and maintained at −4 °C (Fig. 2d,e,f).

### Study design

**Figure 4:**
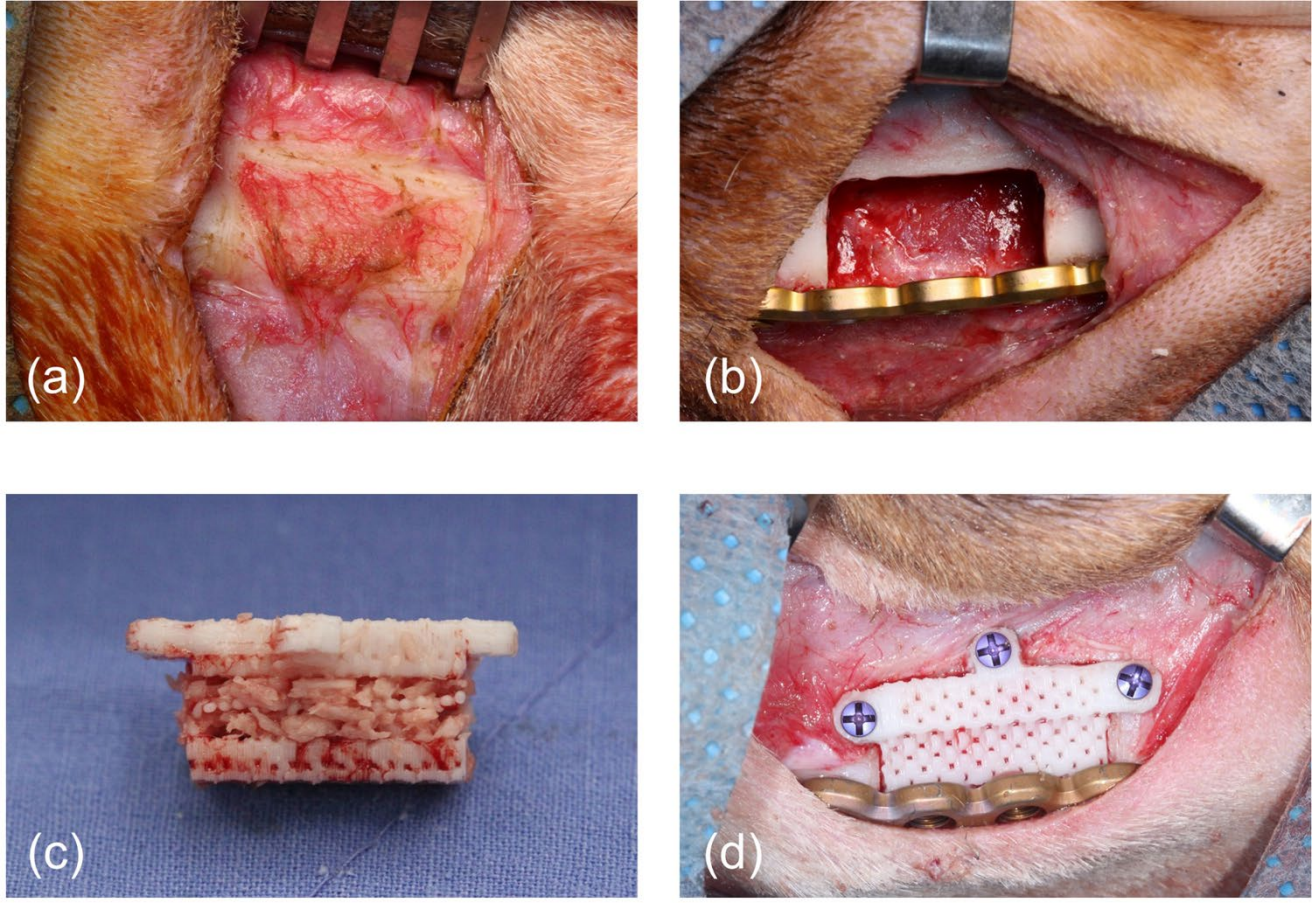
Surgical procedures. (**a**) periosteum left on bone surface to be simultaneously resected, (**b**) a critical-sized defect of mandible in control group, (**c**) PCL/β-TCP scaffold with particularized autogenous bone in PCL/TCP/ABP group, (**d**) implantation of scaffold and reinforcement of mandible with reconstruction plate in PCL/TCP/BMP2 group.

Four experimental groups were constituted at bilateral mandible in eight beagle dogs in this study (n = 4, respectively) as follows: a control group (control, n = 4), PCL/β-TCP scaffold only group (PCL/TCP, n = 4), PCL/β-TCP scaffold + rhBMP-2 50 μg/ml (PCL/TCP/BMP2, n = 4) and PCL/β-TCP scaffold with autogenous bone particles (PCL/TCP/ABP, n = 4) (Table 1). No scaffolds were placed into the defect for the control group. In the PCL/TCP/BMP2 group, at a concentration of 50 μg/ml, rhBMP-2 with a collagen sponge as a carrier was loaded into the scaffolds For the PCL/TCP/ABP group, autogenous bone obtained from mandibular resection was particularized with a bone crusher and inserted into the loose internal structure of scaffolds (Fig. 4c). The sample size was determined by Mead’s resource equation.

**Table 1:** Characteristics of experimental groups (n = 4, respectively).

Group	Composition of scaffold
Control	No scaffold
PCL/TCP	PCL (70 wt%)/β-TCP (30 wt%)
PCL/TCP/BMP2	PCL (70 wt%)/β-TCP (30 wt%)/rhBMP-2 50 μg/ml
PCL/TCP/ABP	PCL (70 wt%)/β-TCP (30 wt%)/autogenous bone particles

### Surgical procedures

Oral prophylaxis was applied to all experimental animals prior to the surgical procedures. The tooth extraction was performed on first molars and premolars in bilateral mandible. After alfaxalone induction (0.2 mg/kg intravenous injection; Hans Nieuwendijk. Nederland), animals were anaesthetized with isoflurane (Choongwae Co., Seoul, Republic of Korea). Then, 1.8 mL of 2% lidocaine HCL with 1:100,000 epinephrine (Huons, Seongnam-si, Gyeonggi-do, Republic of Korea) was injected for infiltrative anesthesia on intraoral mucosa of premolar and molar area. The 4th premolar and 1st molar were sectioned into several parts into mesial and distal roots. After buccal and lingual walls of the extraction socket were preserved intact, the socket was closed with synthetic absorbable suture (3-0 coated VICRYL Plus, Ethicon Inc., NJ, USA) for proper healing. Animals were allowed a two-month recovery period.

2 months from the extraction of the 4th premolar and 1st molar, bone defects were created on bilateral mandible of all 8 beagles. Under general anesthesia as detailed above, local anesthesia was applied on the submandibular skin area. The mandible was exposed via an extraoral submandibular approach. At the level of inferior border in the mandibular body, a rectangular-shaped critical-sized defect (CSD) was outlined with a surgical marking pencil to be 20 mm in length, 10 mm in width, and 10 mm in height (20 × 10 × 10 mm). The en bloc resection of inferior border and periosteum was performed bicortically with reciprocating saw (Fig. 4a). The inferior alveolar neurovascular bundle was ligated. The scaffolds were then implanted into 12 defects in 6 beagles (4 defects in 2 beagles were left empty as negative control) and fixed with 1.95 mm-diameter self-drilling & tapping screws (Optimus Maxillofacial Plating System, Osteonic, Seoul, Republic of Korea) on three interfaces with residual bone (mesial, distal and alveolus). Following the fixation of scaffolds, osteosynthesis was then performed using a 2.4 mm thick titanium reconstruction plate (Optimus Mandible Plating System, Osteonic, Seoul, Republic of Korea) for 2.7 mm-diameter cortical screws (Fig. 4b,d). After copious saline irrigation, the subcutaneous layer was closed with synthetic absorbable suture (3-0 coated VICRYL Plus, Ethicon Inc., NJ, USA). Skin was closed using synthetic non-absorbable monofilament suture (3-0 Dafilon, B.Braun Surgical S.A., Barcelona, Spain). At 12 weeks after implantation, animals were euthanized with intravenous injection of concentrated potassium chloride (Daejung, Sigeung, Korea). After absence of cardiovascular function was verified, block sections including implants, alveolar bone, and surrounding mucosa were collected.

### Micro-computed tomographic analysis

Microcomputed tomographic images were made using a microcomputed tomography scanner (Skyscan 1076; Skyscan, Kontich, Belgium) at a tube voltage of 100 kV and a tube current of 100 μA with rotation steps of 0.5° over a trajectory of 360°. The region of interest (ROI) was defined as a regular cuboid with 1 cm length, 5 mm width and 1 cm height including the internal scaffold space. Image-analysis programs (CTAn 1.12.0.0 and CTVol 2.2.1.0; Skyscan) were used for morphometric analysis and rendering. Mineralized bone volume was calculated by grayscale index from 39 to 52 (defined as radiopaque tissue) in inverse images. Bone volume/tissue volume (BV/TV, mm3), trabecular numbers (Tb.N, 1/mm), trabecular thickness (Tb.Th, mm), and trabecular separation (Tb.Sp, mm) of each group were assessed within ROI. (Supplementary Fig. S1)

### Histological and histomorphometric analysis

Tissues containing the scaffolds were removed en bloc and fixed in 4% neutral-buffered formaldehyde, then dehydrated using an ascending series of alcohol concentrations (80–100%) and embedded in methyl methacrylate (Technovit 7200; Exakt Apparatebau, Norderstedt, Germany) for undecalcified sectioning. The sections containing the whole scaffolds along the sagittal plane were produced at 200 μm using a macro-cutting, then made at a final thickness of 20 μm using a grinding system (Exakt 310 CP series; Exakt Apparatebau, Norderstedt, Germany). The sections were stained with hematoxylin-eosin (H&E) and Goldner’s Masson trichrome separately.

The histologic sections were examined using a light microscope (BX51; Olympus, Tokyo, Japan) to identify newly formed bone (NB), residual scaffold (RS) and nonmineralized tissue (NMT) area under 50× magnification. Area measurements were made using KAPPA ImageBase (Kappa Optronics GmbH, Gleichen, Germany) and Image J (Image J, NIH, USA). The rectangular area of interest (ROI) was defined on the anterior and posterior interfaces between scaffold and residual bone. The ratios of the NB, RS and NMT to the total area were calculated as a percentage.

### Statistical analysis

Quantitative data were expressed as the mean ± standard deviation. Wilcoxon rank-sum test and Kruskal-Wallis test with the Tukey significant difference post hoc test were also used to assess difference among the groups. All analyses were performed using IBM SPSS Statistics for Windows, version 22.0 (IBM Corp., Armonk, NY, USA). A probability value (two-tailed) of less than 0.05 was considered to indicate statistical significance (P < 0.05).

## Results

### Clinical observations

All the animals presented with favorable general status. Except or one PCL/TCP group sample, the remaining 15 samples demonstrated no detectable wound dehiscence or scaffold exposure throughout the experiment. The one scaffold in the PCL/TCP group showed intraoral exposure of the wing structure on the mesial area of defect on postoperative day 7. After intensive care on the intraoral wound for the remaining follow‐up period, no abnormalities were noticed. All the obtained samples were included in the analyses.

### Micro-computed tomographic analysis

**Figure 5:**
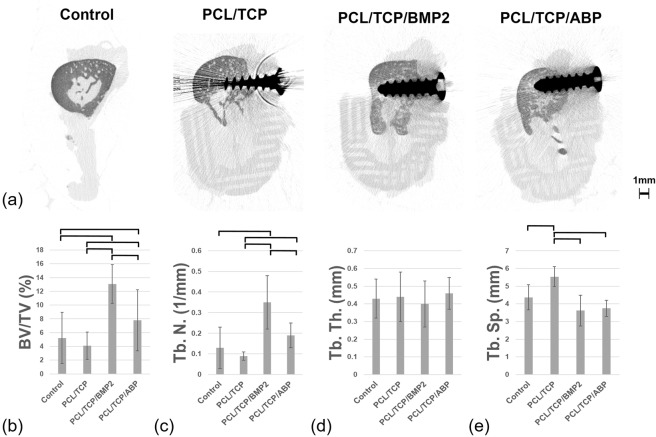
De novo bone formation in micro-computed tomography. (**a**) Coronal view of micro-computed tomography, (**b**) bone volume-total volume ratio (BV/TV), (**c**) trabecular number (Tb. N.), (**d**) trabecular thickness (Tb. Th.), (**e**) trabecular separation (Tb. Sp.). Solid line indicates statistically significant difference between groups.

Volumetric measurement results were obtained using micro-CT (Fig. 5, Supplementary Fig. S2 and Table S1). At 12 weeks after surgery, new bone volumes (mean ± standard deviation, mm3) in the groups were 33.46 ± 21.81, 30.50 ± 16.26, 97.17 ± 28.11 and 68.32 ± 21.47, respectively. The differences between some groups were significant (P < 0.05). Group 3, which was loaded with rhBMP-2, showed the highest new bone volume (mm3).

### Histologic observations and histomorphometric analysis

**Figure 6:**
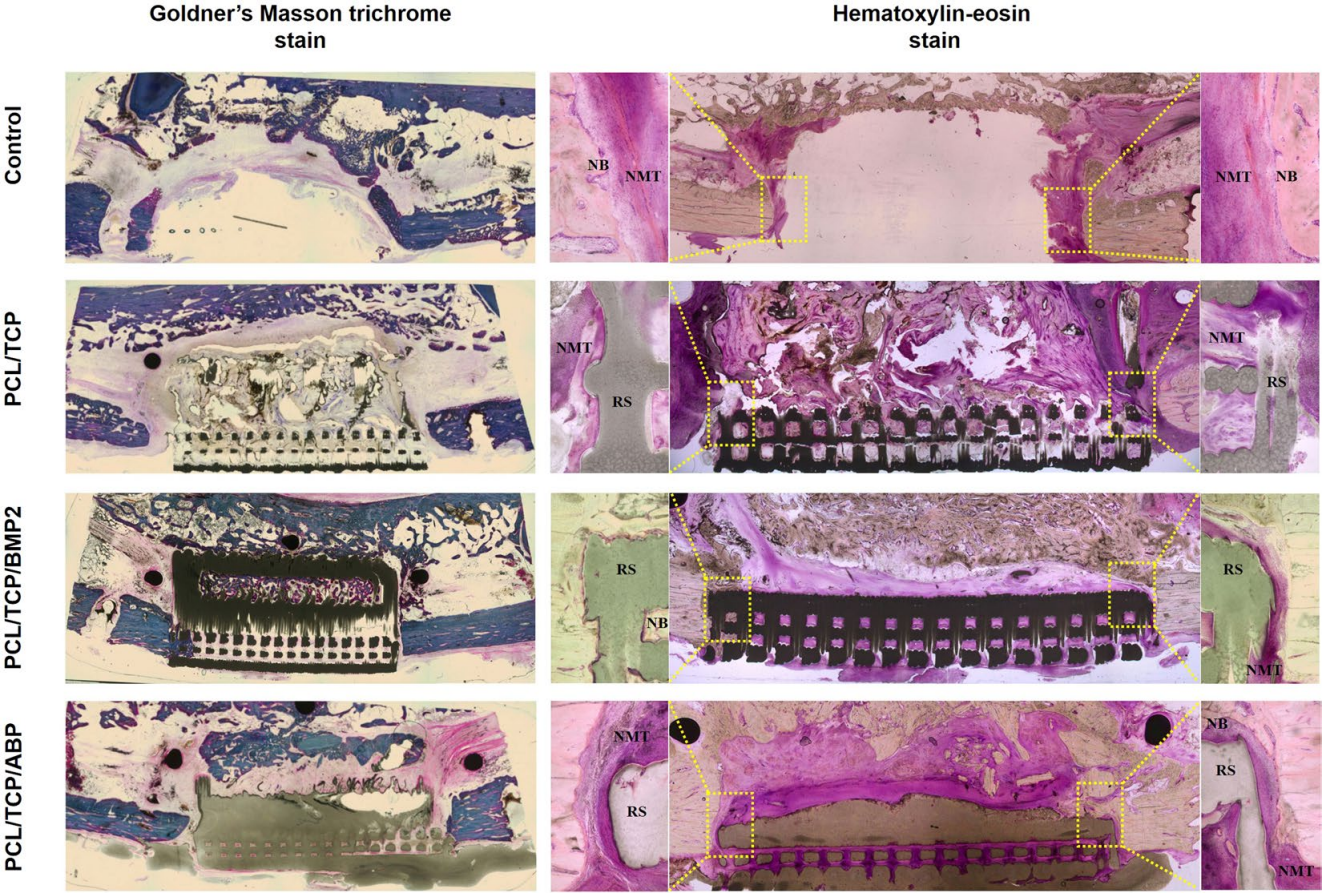
Histologic sections (original magnification 10×). In the left column, the Goldner’s Masson trichrome stained results were shown. The hematoxylin-eosin stain results were presented in right column. ROI was shown in yellow dotted areas and corresponding regions were magnified (100×).

Histological findings are shown in Fig. 6. Scaffolds were properly positioned in mandibular defects. At 12 weeks after surgery, calcification and de novo formation were observed in the experimental groups. In the PCL/TCP group, small amounts of new bone grew into the PCL/β-TCP scaffold and interface region between scaffold and residual bone. More newly formed bone was observed in PCL/TCP/BMP2 and PCL/TCP/ABP groups than in control and PCL/TCP groups. In particular, the loose porous area in the PCL/TCP/BMP2 and PCL/TCP/ABP groups had been filled by new bone and fibrous connective tissues. Abnormal resorption or degradation of the scaffold structure was not observed in any group.

Histomorphometric results are summarized in Table 2. At 12 weeks after surgery, newly formed bone (%) in the control, PCL/TCP, PCL/TCP/BMP2 and PCL/TCP/ABP groups were 22.70 ± 4.24, 25.78 ± 20.94, 33.27 ± 9.64 and 33.32 ± 14.66 respectively. Newly formed bone at interfaces was highest in the PCL/TCP/BMP2 group and lowest in the control group. A trend towards more bone formation was observed in PCL/TCP/BMP2 and PCL/TCP/ABP groups, but the results lacked statistical significance (P = 0.052).

**Table 2:** Histomorphometric analysis within area of interest (n = 4, respectively, mean ± SD).

Parameter	Control	PCL/TCP	PCL/TCP/BMP2	PCL/TCP/ABP	*P value*
NB	22.70 ± 4.24	25.78 ± 20.94	33.27 ± 9.64	33.32 ± 14.66	0.052
(%)					
RS	NA	31.69 ± 7.58	33.31 ± 10.13	32.07 ± 12.14	0.717
(%)					
NMT	77.30 ± 4.24	42.53 ± 14.71	33.41 ± 9.50	34.60 ± 5.64	0.012*
(%)					

Abbreviation: NB, newly formed bone; RS, residual scaffold; NA, not applicable; NMT, nonmineralized tissue

(*p < 0.05).

## Discussion

Our *in vivo* study demonstrated the benefits of 3D-printed PCL/β-TCP scaffolds in mandibular reconstruction. First, the design of scaffold could increase the rigidity of fixation to the residual bone. Previous *in vivo* studies used different fixation modalities to immobilize the scaffold in large defect. Researchers used dental implants^13^, mini screws through the PCL/TCP scaffold^14^ or an endo-prosthetic design of the PCL/TCP scaffold into the mandibular defect of monkey^15^. In this study, we designed less invasive modality with three semicircular shaped, 4.0 mm diameter × 2.0 mm thickness external wing structures containing guide holes for mini-screw fixation. This was to inhibit unpredictable damage to the scaffold, diminution of x-ray scattering during micro-computed tomographic analysis, and to ensure less damage to the adjacent structure and distinct quantification of the de novo bone formation within scaffold. For 12 weeks after implantation of the scaffold, the screw fixations were favorably maintained in the residual bone. Those external wing structures could also be customized for individual geometry of the defect and extended for rigid fixation of mandibular discontinuity defect. Second, MHDS enabled the fabrication of PCL/β-TCP scaffolds with double porous layer. Through the bimodal macro-porosities, we could provide the PCL/β-TCP scaffolds with mechanical support from the cortical bone-like layer of which diameter is approximately 300 μm and enhance the new bone growth from residual bone into the cancellous bone-like layer of which diameter is over 600 μm. The present study revealed acceptable outcomes of new bone growth from the alveolus into the cancellous bone-like layer in the canine mandible. Those new bone that connect both ends of the defect could compensate loss of mechanical strength due to degradation of PCL/β-TCP scaffolds.

The mechanical property of PCL/β-TCP scaffold might be insufficient to be applied alone in the reconstruction of human mandible. According to Bao *et al*., the hydroxyapatite/polycaprolactone (HA/PCL) scaffolds were fabricated with biomimetic structure of which proper osseointegration and mechanical property was observed in rabbit tibia^16^. They used titanium miniplate fixation to stabilize the HA/PCL scaffolds in the weight bearing bone. Similar to the mechanical property of the HA/PCL scaffolds, the compressive strength of PCL/β-TCP scaffolds has been verified as approximately 20 MPa, which is 2 times higher than that of cancellous bone and one tenth of cortical bone in mandible^17,18^ (Material, methods and results are detailed in Supplementary Discussion S1 and Supplementary Fig. S3). In the clinical situation, reconstructive surgeons should consider the biomechanics of mandible including tension, compression and torsion^18^. For distribution of loading forces to the mandible, the titanium plate with load-bearing fixation method should be combined for mandibular reconstruction using 3D-printed PCL/β-TCP scaffolds.

The classic definition of CSD is the smallest size defect that will not heal over the lifetime of the animal or a defect which shows less than 10 percent bone regeneration during the first year of defect healing^19–22^. Defects larger than CSD result in non-healing with bone regeneration and fibrous tissue engaging in the defects. To date, only a few studies have documented the CSD in the canine mandible^23,24^. According to Huh *et al*., after removal of the periosteum in mongrel dogs, bony bridges were not observed in mandibular defects more than 15 mm size at 6 months after segmental mandibulectomy. They suggested the critical-sized defect in canine mandible is 50 mm in presence of periosteum and 15 mm in absence of periosteum. We performed the mandibular resection including periosteum with size of 20 × 10 × 10 mm to create a CSD in beagle dogs. Previous studies for PCL/TCP scaffold used similar dimensions as a CSD in canine mandible. Rai *et al*. created a CSD of 18 × 10 × 7 mm in frontal and caudal mandible of mongrel dogs^13^. Using CSD of 20 × 10 × 10 mm in buccal cortical plate of canine mandible, A. Khojasteh *et al*. reported the bone regeneration of PCL/TCP scaffold with BMSC^14^.

In this study design, there was a need to limit the use of rhBMP-2. Since the potential carcinogenicity of rhBMP-2 in association with oral squamous cell carcinoma (OSCC) remains controversial^25–29^. Gao *et al*. suggest that BMP-2 may not have adverse effects on OSCC, as measured by proliferation and angiogenesis^25^. However, human OSCC cell lines have been shown to express BMP-2 and be associated with rapid tumor growth in orthotopic animal model^27^. Although there is no agreement whether rhBMP-2 promotes, inhibits, or has no association with tumorigenesis of OSCC, maxillofacial surgeons should be cautious in applying rhBMP-2 in malignant tumor patients. In clinical situations, a PCL/β-TCP scaffold combined with autogenous bone particle is applicable for mandibular defect after malignant tumor resection. Meanwhile, a rhBMP-2 loaded PCL/β-TCP scaffold can be applied in mandibular defect derived from trauma, osteomyelitis, medication-related osteonecrosis of the jaw and particular benign lesions (ameloblastoma, odontogenic keratocyst, giant cell granuloma and etc.)^2^.

Our study has several limitations. The volume of new bone formation was insufficient for clinical application in mandibular reconstruction. In the present study, we also used the periosteal resection model to evaluate the efficacy of PCL/β-TCP scaffold with low dose of rhBMP-2. Although the PCL/TCP/BMP2 group showed no significant difference in the volume of new bone formation compared with PCL/TCP/ABP groups, the absolute volume of new bone formation was remarkably lower than that of previous studies. To exclude the healing potential of periosteum and assess the bone regeneration efficacy of scaffolds, previous studies reported *in vivo* results in a periosteal resection model. In the absence of periosteum, researchers documented that bone regeneration with scaffold alone might be challenging^30–32^. To achieve clinically acceptable results, further studies are needed to determine the adequate concentration of rhBMP-2 for periosteal resection model in mandibular discontinuity defect. Another limitation is that degradation period of PCL/β-TCP scaffold could not be assessed at 12 weeks after surgery. Previous studies demonstrated initiation of *in vitro* degradation within 28 days in simulated body fluids^33^, remarkable *in vivo* degradation at 24 weeks after implantation in rat abdomen^34^, and about 33% *in vivo* degradation of PCL/β-TCP scaffold from 6 to 9 months after implantation in canine mandible^13^. More extensive investigation is necessary for long term observation on degradation of scaffold and associated inflammatory response.

This study was conducted to evaluate the feasibility of 3D-printed PCL/β-TCP scaffolds in large defect of canine mandible. The scaffold incorporated heterogeneous pore sizes for rapid bone ingrowth and additional wing structures for more stable screw fixation. Within the limitations of the present study, 3D-printed PCL/β-TCP scaffolds showed acceptable potential for mandibular reconstruction. For future clinical implementation, a larger study of PCL/β-TCP scaffolds with high dose of growth factors is required.

## Supplementary information

Supplementary information.
