# Supplementary material for: Efficacy of three-dimensionally printed polycaprolactone/beta tricalcium phosphate scaffold on mandibular reconstruction
Source: Sci Rep. 2020 Mar 18;10:4979. doi: 10.1038/s41598-020-61944-w (PMC7080805; doi:10.1038/s41598-020-61944-w)
Supplement: Supplementary file 1 — Supplementary information. [file 41598_2020_61944_MOESM1_ESM.pdf]

Supplementary Information

Article in *Scientific Reports*

Efficacy of three-dimensionally printed polycaprolactone/beta tricalcium phosphate scaffold on mandibular reconstruction

Sanghoon Lee, Dami Choi, Jin-Hyung Shim, Woong Nam

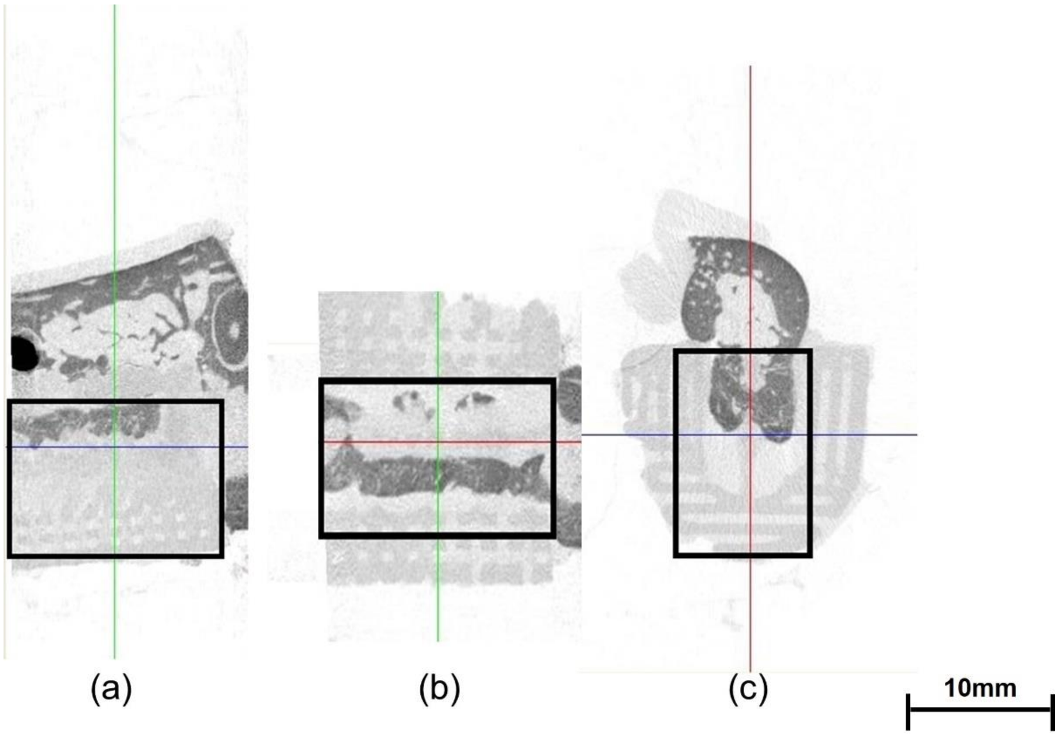

Supplementary figure S1. Region of interest (ROI) in micro-computed tomographic analysis. ROI (length: 20 mm, width: 10mm and height: 10 mm) is shown in (a) sagittal view, (b) axial view (c) coronal view.

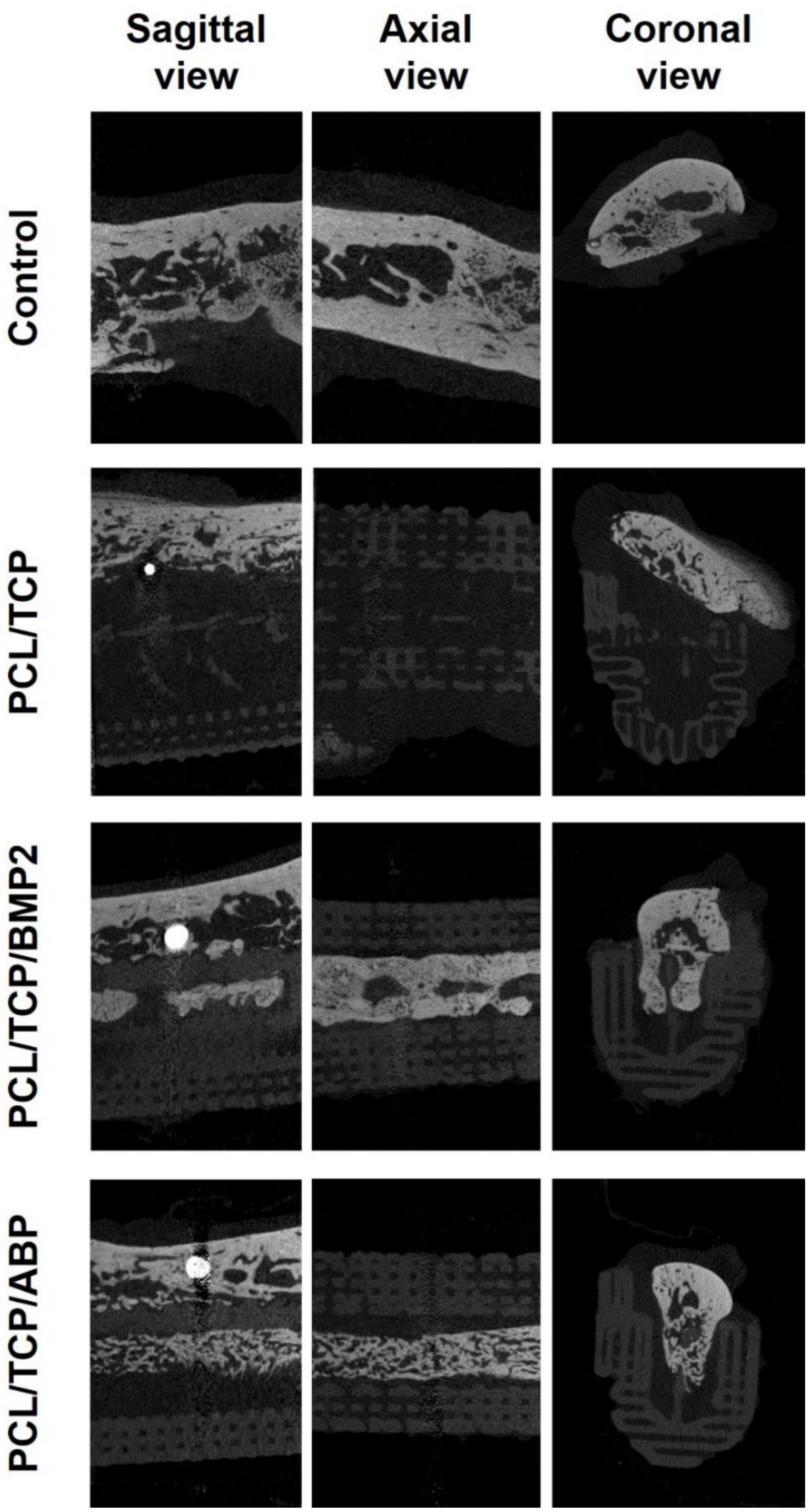

Supplementary figure S2. De novo bone formation in micro-computed tomography.

Supplementary table S1. The entire list of samples in micro computed tomographic analysis (n=4, respectively)

Group		BV [mm^3]	TV [mm^3]	BV/TV [%]	Tb.N. 1/mm]	Tb. Th. [mm]	Tb.Sp. [mm]
Control	1L_1	21.72192	611.7652	3.5507	0.06192	0.57347	4.03022
Control	1L_2	10.66517	611.7652	1.74334	0.04723	0.36911	4.9486
Control	2L_1	18.96104	611.7652	3.0994	0.06263	0.4949	4.77029
Control	2L_2	71.92643	539.97055	13.32044	0.28017	0.47544	3.9391
Control	4R_1	14.93379	611.7652	2.4411	0.04962	0.49198	4.99995
Control	4R_2	30.14735	546.79385	5.51348	0.11799	0.46728	5.06816
Control	6L_1	42.38388	819.26879	5.17338	0.15506	0.33364	4.14795
Control	6L_2	56.9055	819.26879	6.94589	0.29726	0.23367	3.04188
PCL/TCP	1R_1	13.46684	612.99611	2.19689	0.07887	0.27855	4.80862
PCL/TCP	1R_2	36.3104	732.11132	4.95968	0.1166	0.42536	5.81638
PCL/TCP	2R_1	20.15944	801.44366	2.51539	0.06693	0.37582	6.41715
PCL/TCP	2R_2	22.74134	685.069	3.31957	0.10367	0.32021	5.20456
PCL/TCP	3L_1	64.00712	781.50055	8.19028	0.12462	0.65721	4.7855
PCL/TCP	3L_2	31.74782	743.73231	4.26872	0.07423	0.57509	6.00437
PCL/TCP	5L_1	38.30764	793.12155	4.82998	0.08506	0.56785	5.72631
PCL/TCP	5L_2	17.30743	711.77457	2.43159	0.07347	0.33094	5.52537
PCL/TCP/BMP2	5R_1	128.32014	799.05877	16.05891	0.54488	0.29621	2.52365
PCL/TCP/BMP2	5R_2	140.3853	807.64779	17.382	0.48172	0.36155	2.61632
PCL/TCP/BMP2	6R_1	78.48574	611.7652	12.82939	0.212	0.60515	4.70454
PCL/TCP/BMP2	6R_2	76.7043	611.7652	12.53819	0.24368	0.51454	4.42503
PCL/TCP/BMP2	7L_1	62.18892	751.29347	8.27758	0.31038	0.2667	3.34374
PCL/TCP/BMP2	7L_2	77.435	697.24833	11.1058	0.46524	0.23871	2.92949
PCL/TCP/BMP2	8L_1	114.74849	819.26879	14.00621	0.29146	0.48055	3.9594
PCL/TCP/BMP2	8L_2	99.09021	805.56708	12.30068	0.26081	0.47164	4.4304
PCL/TCP/ABP2	3R_1	73.98472	755.35331	9.79472	0.20608	0.47529	3.4649
PCL/TCP/ABP	3R_2	85.24843	819.26879	10.40543	0.24056	0.43255	3.2822
PCL/TCP/ABP	4L_1	61.89279	761.16381	8.13134	0.13228	0.6147	4.38327
PCL/TCP/ABP	4L_2	88.88462	819.26879	10.84926	0.20777	0.52217	3.19869
PCL/TCP/ABP	7R_1	53.01299	746.63756	7.10023	0.14667	0.4841	4.13261
PCL/TCP/ABP	7R_2	45.30782	703.99763	6.43579	0.13504	0.47657	3.62104
PCL/TCP/ABP	8R_1	98.06389	819.26879	11.96968	0.28907	0.41408	3.64635
PCL/TCP/ABP	8R_2	40.13849	819.26879	4.89931	0.1614	0.30354	4.29061

Supplementary Discussion S1

Material and methods

Compressive strength test was carried out with block-shaped specimens of 10*4*10mm in length, width and height, respectively, which were tested according to ISO604 - Plastics — Determination of compressive properties.

Results

Tests on PCL/β-TCP scaffold (8:2) that is identical composition and porous structure as *in vivo* experiment revealed that the mean compressive strength was 20.85 (SD = ± 1.25) Mpa (n = 3, Supplementary Figure S3). The compressive strength of our PCL/β-TCP scaffold is 2 times higher than that of the cancellous bone and one tenth of cortical bone in mandible [1,2].

REFERENCES

1. Hannink, G. & Arts, J. J. Bioresorbability, porosity and mechanical strength of bone substitutes: what is optimal for bone regeneration? *Injury* **42 Suppl 2**, S22-25 (2011).
2. Misch, C. E., Qu, Z. & Bidez, M. W. Mechanical properties of trabecular bone in the human mandible: implications for dental implant treatment planning and surgical placement. *J Oral Maxillofac Surg* **57**, 700-706; discussion 706-708 (1999).

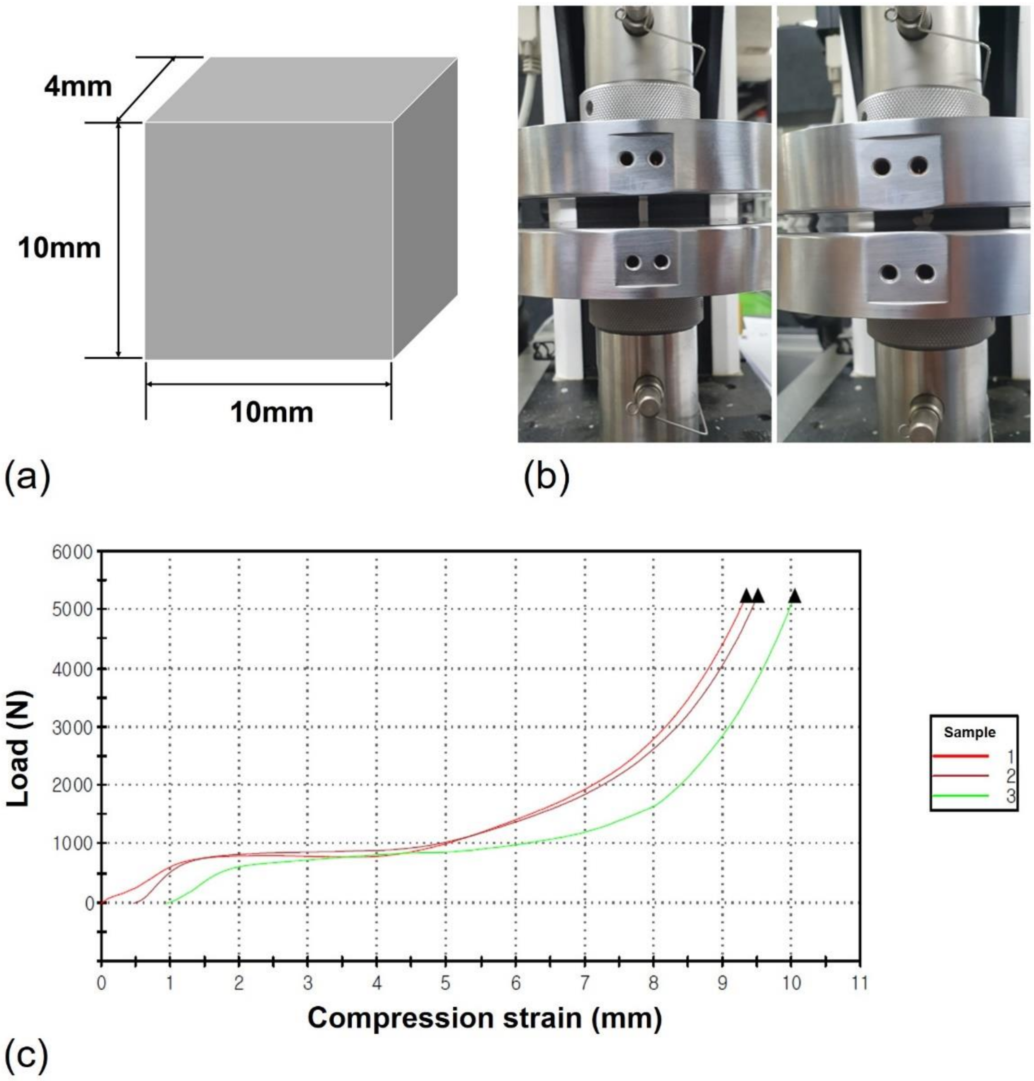

Supplementary figure S3. Compression test of 3D-printed PCL/ β-TCP scaffolds according to ISO604 - Plastics — Determination of compressive properties. (a) dimension of experimental sample, (b) photograph of experiment, (c) result of compression test. (n=3)
